# Exogenous brassinolide improves the antioxidant capacity of *Pinellia ternata* by enhancing the enzymatic and nonenzymatic defense systems under non-stress conditions

**DOI:** 10.3389/fpls.2022.917301

**Published:** 2022-07-25

**Authors:** Chenchen Guo, Ying Chen, Mengyue Wang, Yu Du, Dengyun Wu, Jianzhou Chu, Xiaoqin Yao

**Affiliations:** ^1^School of Life Sciences, Hebei University, Baoding, China; ^2^Institute of Life Sciences and Green Development, Hebei University, Baoding, China; ^3^Key Laboratory of Microbial Diversity Research and Application of Hebei Province, Baoding, China

**Keywords:** antioxidant enzyme, ascorbic acid, brassinolide, glutathione, flavonoid

## Abstract

Brassinolide (BR) improves the antioxidant capacity of plants under various abiotic stresses. However, it is not clear about the effect of BR on the antioxidant capacity in plants under non-stress conditions. In the present study, the antioxidant defense response of *Pinellia ternata* was to be assessed by applying BR and propiconazole (Pcz) under non-stress conditions. BR treatment enhanced the flavonoid content, peroxidase, and ascorbate peroxidase (APX) activity by 12.31, 30.62, and 25.08% and led to an increase in 2-diphenyl-1-picrylhydrazyl (DPPH) radical scavenging activity by 4.31% and a decrease in malondialdehyde content by 1.04%. Exogenous application of BR improved the expression levels of PAL, CHS, CHI, and DFR genes by 3. 18-, 3. 39-, 2. 21-, and 0.87-fold in flavonoid biosynthesis, PGI, PMI, and GME genes by 6. 60-, 1437. 79-, and 3.11-fold in ascorbic acid (ASA), biosynthesis, and γECs and GSHS genes by 6.08- and 2.61-fold in glutathione (GSH) biosynthesis pathway, and the expression of these genes were inhibited by Pcz treatment. In addition, BR treatment promoted the ASA–GSH cycle by enhancing the expression of APX, DHAR, and MDHAR genes, which were enhanced by 3. 33-, 157. 85-, and 154.91-fold, respectively. These results provided novel insights into the effect of BR on the antioxidant capacity in bulbil of *P. ternata* under non-stress conditions and useful knowledge of applying BR to enhance the antioxidant capacity of plants.

## Introduction

Brassinolide (BR) is a phytohormone that is essential for plant growth and development (Kaya et al., 2019). It was found that exogenous application of BR improved plants’ tolerance to abiotic stress (Khan et al., 2022). Environmental changes and anthropogenic activities have caused many abiotic stresses to plants, such as water stress, temperature stress, salinity stress, heavy metals stress, UVB and UVA radiation stress, organic pollutants stress, etc., which greatly affect plant growth and development, ultimately leading to reduced plants yields and quality and limiting the sustainability of agriculture (Hasanuzzaman et al., 2020). When changes in the environmental factors exceed the limits of plant tolerance, these factors become stressors to plant growth (Ortiz-Espín et al., 2017). BR improving plant antioxidant capacity is an effective adaptive mechanism for plants to iron deficiency stress, heavy metal stress, low-temperature stress, water stress, drought stress, etc. (Kaya et al., 2020a; Faizan et al., 2021; Sun et al., 2022). Kaya et al. (2020b) reported that brassinosteroid treatment reduced oxidative stress-related parameters by increasing dehydroascorbate reductase (DHAR), ascorbate peroxidase (APX), glutathione reductase (GR), and nitrate reductase activities of pepper under cadmium stress. BR maintains the normal growth of tomatoes by increasing antioxidant capacity under Cr (VI) metal stress (Jan et al., 2020). The epi-brassinolide treatment increased the activity of GR, APX, dismutase (SOD), and catalase (CAT), and the contents of flavonoids, total phenols, ascorbic acid (ASA), and glutathione (GSH) in tomatoes to effectively combat salinity stress (Ahanger et al., 2020). Kaya et al. (2019) have also demonstrated that BR treatment improved pepper to water stress by inducing NO generation and enhancing CAT, and POD activities, thus, reducing the malondialdehyde (MDA) and H_2_O_2_ content. It is found that abiotic stress disrupts intracellular homeostasis and leads to an increase in reactive oxygen species contents (ROSs), such as O_2_^–^ and H_2_O_2_ (Faizan et al., 2021; Siddiqi and Husen, 2021). The accumulation of excessive ROS leads to oxidative damage to carbohydrates, proteins, DNA, lipids, and other macromolecules and promotes apoptosis and aging (Raja et al., 2017). Thus, BR treatment improves the antioxidant capacity to eliminate the excessive accumulation of ROSs. The molecular and physiological functions of BR have been well understood under stressful conditions (Hafeez et al., 2021). Although this research field has aroused great interest, it is not clear whether BR treatment regulates the antioxidant capacity of plants under non-stress conditions. Propiconazole (Pcz) is a specific inhibitor of BR biosynthesis in maize, *Arabidopsis*, and soybean (Hartwig et al., 2012; Song et al., 2019). Furthermore, it has not yet been elucidated how the antioxidant system responds to Pcz treatment under non-stress conditions.

Harmful environmental conditions induce the accumulation of ROS in plant cells. Moreover, low levels of ROS are signals for plants to respond to environmental changes. If the strength of the environmental stress surpasses the antioxidant capacity of the plant cell, it results in an intracellular redox imbalance. There are enzymatic and non-enzymatic antioxidant defense systems in plants, which maintain the balance of the antioxidant defense system and ROS accumulation. The enzymatic defense systems are mainly composed of different enzymes, such as DHAR, APX, GR, monodehydroascorbate reductase (MDHAR), SOD, CAT, and peroxidase (POD) (Ahmad et al., 2010; Basit et al., 2022). In non-enzymatic antioxidant defense, the main components of plant response to oxidative stress are flavonoids, ASA, and GSH. It is well known that abiotic stresses affect plant growth and development. Therefore, there is increasing attention to studying the physiological and molecular mechanisms that enhance plant tolerance to abiotic stress. Understanding plant responses to stress and improving plant tolerance is important to alleviate the effects of changes in environmental factors on crops.

*Pinellia ternata* is a perennial medicinal plant and is widely found in Eastern Asia, especially in China. In China, *P. ternata* is being used to treat various diseases such as vomiting, cough, traumatic injury, and inflammation (Lu et al., 2020). The demand for *P. ternata* is gradually increasing worldwide. Due to environmental changes and anthropogenic activities, the yield of artificial cultivation is low and cannot meet the market demand. Our previous results showed that exogenous application of BR (0.10 mg l^–1^) BR increased the yield, total flavonoid content, and ascorbic acid content in bulbil of *P. ternata* by 78.70, 13.77, and 8.04% (Guo et al., 2021). Therefore, it is necessary to further investigate the response of enzymatic and non-enzymatic antioxidant capacity of *P. ternata* to BR under non-stress conditions, which contributes to a better understanding of the environmental tolerance of *P. ternata* under artificial cultivation conditions.

In recent years, transcriptome sequencing has become an effective method for exploring the molecular mechanisms of different metabolic pathways of plants (Zhang et al., 2019).

Based on the transcriptome sequences and physiological data, the roles of BR on the enzymatic and non-enzymatic antioxidant capacity systems in bulbil of *P. ternata* under non-stress conditions were revealed. These results provided a reference for understanding the regulation of BR on antioxidant capacity in bulbil of *P. ternata* under non-stress conditions and useful knowledge of applying BR to improve the plant tolerance to abiotic stresses. We hypothesize that applying BR increases the antioxidant capacity by enhancing the enzymatic and non-enzymatic defense systems under non-stress conditions.

## Materials and methods

### Plant material and treatments

Seed bulbs of *P. ternata* were sown in pots containing humus soil. After three-leaf expanded, the control, BR-treated, and Pcz-treated plants were sprayed through the foliage with distilled water, 0.1 mg l^–1^ BR, and 1 μM Pcz every day, respectively. Tween 20 (0.02%, v/v) as a surfactant was mixed with the distilled water, BR, and Pcz solution. Pots were maintained in a completely randomized design placed under indoor conditions with four replicates for each treatment. Tuber and bulbil samples were collected at the bulbil expansion stage and snap-frozen using liquid nitrogen until RNA extraction and physiological indexes determination.

### Measurements of flavonoid, ascorbic acid, glutathione, and malondialdehyde

The levels of flavonoid and ASA in bulbil were measured by the AlCl_3_ colorimetry and 24-dinitrophenylhydrazine method (Xue et al., 2021). Flavonoid and ASA content were presented as mg g^–1^ dried weight (DW) and mg g^–1^ fresh weight (FW), respectively. The levels of GSH in bulbil were determined as described previously (Krivosheeva et al., 1996) and were indicated as mg g^–1^ FW. MDA contents were measured by the thiobarbituric acid method and were calculated as described by Shi et al. (2017).

### Measurements of peroxidase, superoxide dismutase, glutathione reductase, ascorbate peroxidase, and DPPH radical activity

The activity of SOD was measured by the method of Ghassemi-Golezani et al. (2020) and was indicated as U g^–1^. The activity of POD was measured by the method of Yuan et al. (2017) and was indicated as U g^–1^ min^–1^. DPPH radical activity was extracted from a dried sample with ethanol absolute and was calculated as described by Xue et al. (2021). The APX activity was performed based on a literature procedure (Nakano and Asada, 1981) and was indicated as U g^–1^ min^–1^. The activity of GR was measured by the method of Krivosheeva et al. (1996) and was presented as U g^–1^ min^–1^.

### RNA isolation, cDNA library preparation, and sequencing

For RNA-Seq and qRT–PCR, RNA samples were prepared using three replicates of bulbil from each treatment. We used the RNA extraction kit (Cowin Biosciences, Beijing) to extract total RNA and checked the quality and quantity of RNA by NanoDrop2000, Agilent2100 Nano, and RNase-free agarose gel electrophoresis. The Illumina Truseq™ RNA sample preparation kit technology was used to prepare RNA-Seq libraries and the libraries were sequenced on an Illumina Nova seq 6000 platform.

### Transcriptome assembly and functional annotation

After sequencing, the clean data were obtained by using the software Seqprep and sickle. We used Trinity software for clean data assembly, and the assembly results were evaluated and optimized using transrate and Busco softwares. We used the software of HMMER3 and DIAMOND to compare the assembled unigenes database with the NR, Pfam, Swiss-Prot, and COG and obtain functional annotations of unigenes by comparing the protein with the highest sequences similarity. In addition, the functional annotations and biological pathway analysis of unigenes were also performed in the KEGG database and GO database using Kobas and Blast2gO software.

### Differentially expressed genes analysis

The DEGs after BR and Pcz treatments were analyzed using DEseq2. TPM (transcripts per kilobase per million mapped reads) was used to quantify gene expression. We used a *P* < 0.05 and | log2 (fold change)| ≥2 as the threshold for determining the significant differences between treatment and control samples. The significant enrichment terms of DEGs on the GO and KEGG database were calculated with adjusted *p*-values (FDR) <0.05. Subsequently, we screened unigenes participating in the flavonoid, ASA, GSH biosynthesis pathways, and ASA-GSH cycle from the functional annotation results and generated heatmaps of expression levels of these genes.

### qRT–PCR analysis of filtered genes

In total, twenty-seven differentially expressed flavonoid, ascorbic acid, glutathione, and ASA–GSH metabolism-related unigenes were selected for qRT–PCR analysis. The reverse transcription kit (Vazyme, Nanjing, China) reverse transcribes 1 μg of total RNA into cDNA for qPCR. The qRT–PCR reactions were carried out using a 20 μl reaction volume containing Chamq universal SYBR qPCR master mix (Vazyme, Nanjing, China), primer, cDNA template, and ddH_2_O and were performed on a LightCycle 96 system (Roche, Switzerland). All the primers of unigenes were designed online by the Integrated DNA Technologies website (Supplementary Table 1). The relative expression levels of unigenes were calculated by the 2^–Δ^ ^Δ^ *^CT^* method using the 18s gene of *P. ternata* as a reference gene (Xue et al., 2019).

### Statistical analysis

The physiological data were expressed as mean values representing four biological replicates ± standard error. The physiological data were performed with the software Statistical Package for Social Science (SPSS, version 26.0). Homogeneity of variance was tested using the Levene test before analysis. Results were statistically analyzed using one-way ANOVA, followed by Tukey’s test to ascertain whether they were significantly different (*p*<0.05).

## Results

### Effects of applying brassinolide on flavonoid, ascorbic acid, and glutathione contents

**FIGURE 1:**
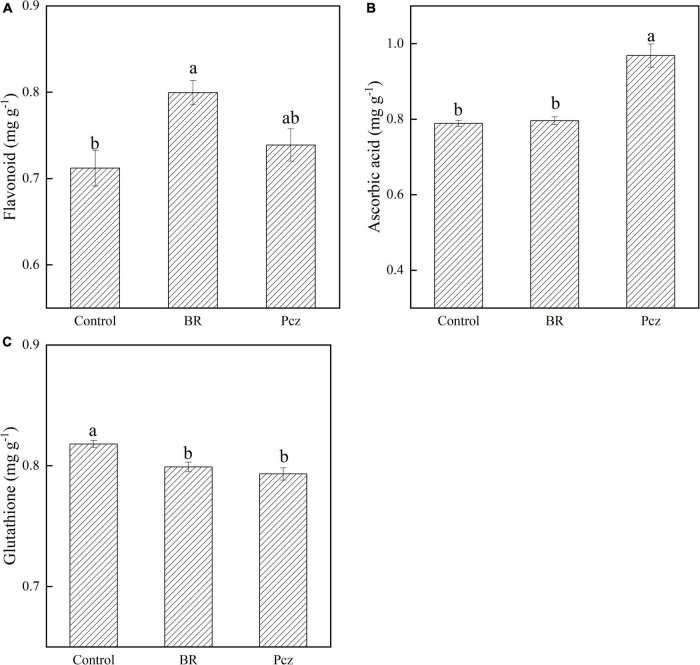
Flavonoid **(A)**, ascorbic acid **(B)**, and glutathione **(C)** content in bulbil of *P. ternata* under BR and Pcz treatments. The bars with different letters are significantly different from each treatment (*p* < 0.05). Values are means of four replicates ± SE.

Application of BR resulted in an increase of 12.31% in the flavonoid content of bulbil (Figure 1). The effect of Pcz treatment on the flavonoid content was not significantly different compared with the control. The ASA content was improved by 22.77% after the Pcz application. There was no significant difference in ascorbic acid content between BR treatment and control. BR and Pcz treatments decreased the GSH content in bulbil by 2.32 and 3.03%.

### Effects of brassinolide treatment on peroxidase, superoxide dismutase, glutathione reductase, and ascorbate peroxidase activities

**FIGURE 2:**
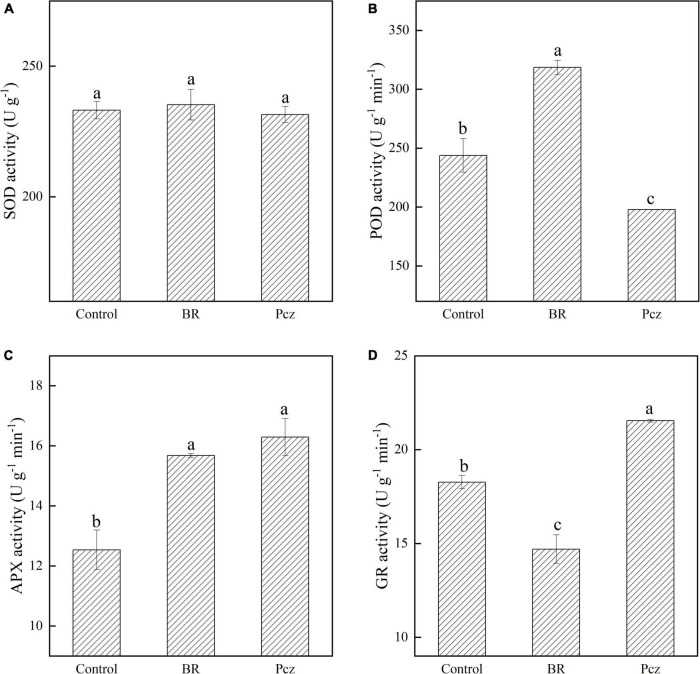
Superoxide dismutase [SOD **(A)**], peroxidase [POD **(B)**], ascorbate peroxidase [APX **(C)**], and glutathione reductase [GR **(D)**] activity in bulbil of *P. ternata* under BR and Pcz treatments. The bars with different letters are significantly different from each treatment (*p* < 0.05). Values are means of four replicates ± SE.

There was no significant effect of the BR and Pcz application on the SOD activity (Figure 2A). The POD activity was enhanced by 30.62% after the BR application but was decreased by 18.87% after the Pcz application (Figure 2B). Application of BR and Pcz enhanced the APX activity by 25.08 and 29.95% (Figure 2C). In addition, the GR activity was reduced by 19.56% after the BR application but was raised by 17.91% after the Pcz application (Figure 2D).

### Effects of brassinolide treatment on DPPH radical activity and malondialdehyde contents

**FIGURE 3:**
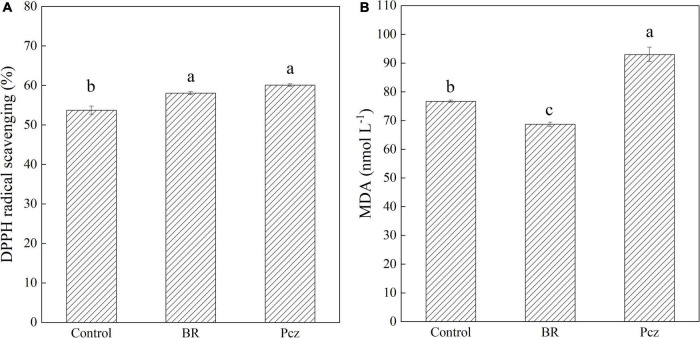
DPPH radical scavenging activity **(A)**, and malondialdehyde content [MDA **(B)**] in bulbil of *P. ternata* under BR and Pcz treatments. The bars with different letters are significantly different from each treatment (*p* < 0.05). Values are means of four replicates ± SE.

The DPPH radical scavenging was improved in BR and Pcz treatments by 4.31% and 6.37%, respectively (Figure 3A). The MDA content was reduced in the application of BR by 1.04% but was enhanced in the application of Pcz by 2.13% (Figure 3B).

### RNA-seq and assembly of unigenes

To investigate the transcriptome response to BR induction in the bulbil of *P. ternata*, the Illumina Nova seq 6000 platform was used to sequence nine cDNA libraries constructed from high-quality RNA. These libraries produced 57,117,606, 52,536,920, 53,506,752, 53,506,752, 50,190,552, 62,617,020, 55,852,426, 55,006,196, and 54,818,536 clear reads, respectively. The Q30 (sequencing error rate <0.1%) was at least 94.25%. Subsequently, we obtained 115,445 unigenes with an N50 value of 1,189 bp by assembling clean reads. The length range of these unigenes was 201 to 18,826 bp, with an average length of 754 bp (Table 1).

**TABLE 1:** Length distribution of assembled unigenes.

Length	Number of unigenes	Percent of unigenes
200∼500	67107	58%
501∼1000	25955	22%
1001∼1500	8788	8%
1501∼2000	5054	4%
2001∼2500	3291	3%
2501∼3000	2118	2%
3001∼3500	1255	1%
3501∼4000	844	1%
4001∼4500	523	0%
> 4500	1054	1%

### Function annotation and classification

**FIGURE 4:**
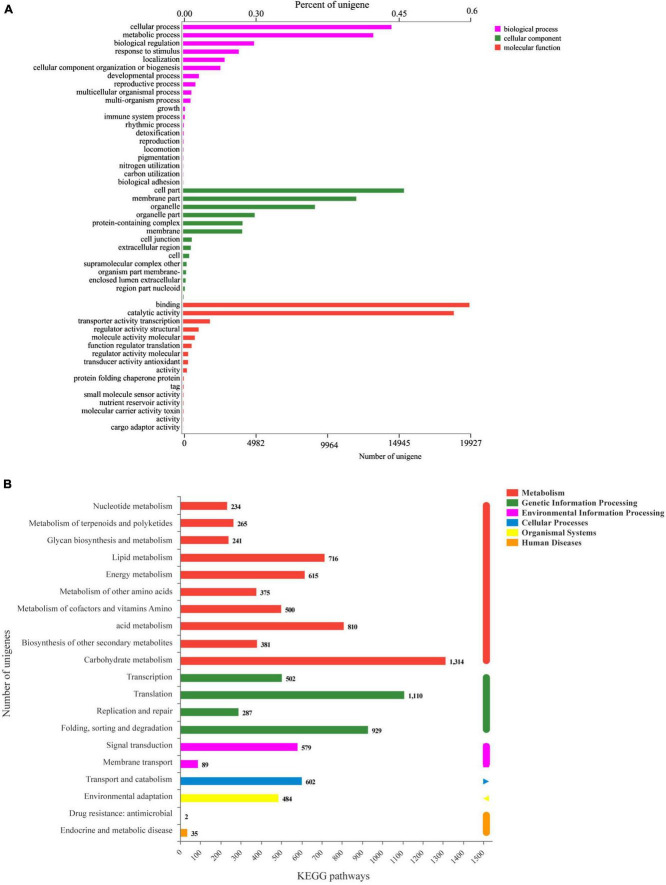
Functional annotations of the unigenes of *P. ternata* bulbil transcriptome. **(A)**, GO function annotation. **(B)**, KEGG function annotation.

A total of 40,992 unigenes (35.51%) were annotated in the six databases. For GO annotation, the main categories were “cellular process,” “metabolic process,” “cell part,” “membrane part,” “binding,” and “catalytic activity” (Figure 4A). The main classification of KEGG was assigned to “carbohydrate metabolism,” “translation,” “folding, sorting, and degradation,” “acid metabolism,” etc. (Figure 4B).

### Comparative analysis of differentially expressed genes

**FIGURE 5:**
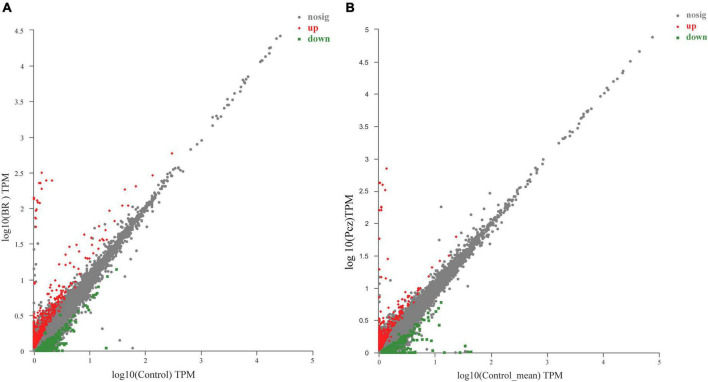
Identification of differential expression genes (DEGs) among control, BR, and Pcz treatments. **(A)**, scatter diagram of DEGs in control and BR groups. **(B)**, scatter diagram of DEGs in control and Pcz groups.

**FIGURE 6:**
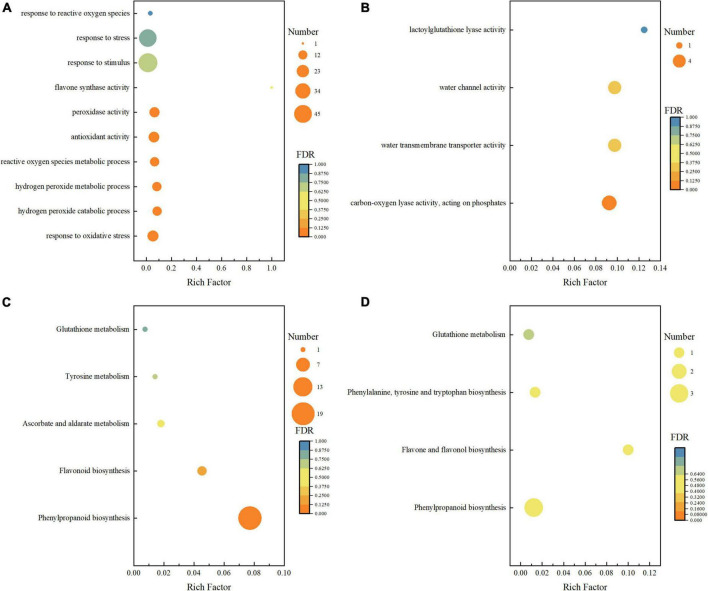
Enrichment analyses of differential expression genes among control, BR, and Pcz. **(A)**, GO enrichment analyses in control and BR groups. **(B)**, GO enrichment analyses in control and PCZ groups. **(C)**, KEGG enrichment analyses in control and BR groups. **(D)**, KEGG enrichment analyses in control and Pcz groups.

A total of 818 (476 upregulated and 342 downregulated) and 697 (389 upregulated and 308 downregulated) unigenes were differentially expressed in BR-treated and Pcz-treated groups (Figure 5). To further investigate the DEGs in the BR-treated and Pcz-treated groups, we performed GO and KEGG pathway enrichment analysis. The results showed that the higher enrichment groups of GO were a response to oxidative stress (GO: 0006979), hydrogen peroxide catabolic process (GO: 0042744), reactive oxygen species metabolic process (GO: 0072593), antioxidant activity (GO: 0016209), flavone synthase activity (GO: 0033759), hydrogen peroxide metabolic process (GO: 0042743), peroxidase activity (GO: 0004601), response to stress (GO: 0006950), response to stimulus (GO: 0050896), and response to reactive oxygen species (GO: 0000302) in BR-treated groups, and were carbon–oxygen lyase activity (GO: 0016838), and lactoylglutathione lyase activity (GO: 0004462). in Pcz-treated groups (Figures 6A,B). KEGG enrichment analysis showed that the terms of phenylpropanoid biosynthesis (map00940), flavonoid biosynthesis (map00941), ascorbate and aldarate metabolism (map00053), tyrosine metabolism (map00350), and glutathione metabolism (map00480) were enriched under BR treatment, and the terms of phenylpropanoid biosynthesis (map00940), flavone and flavanol biosynthesis (map00944), phenylalanine (map00400), and glutathione metabolism (map00480) were enriched under Pcz treatments, compared to the control, respectively (Figures 6C,D).

### Differentially expressed genes involved in the flavonoid biosynthesis

**FIGURE 7:**
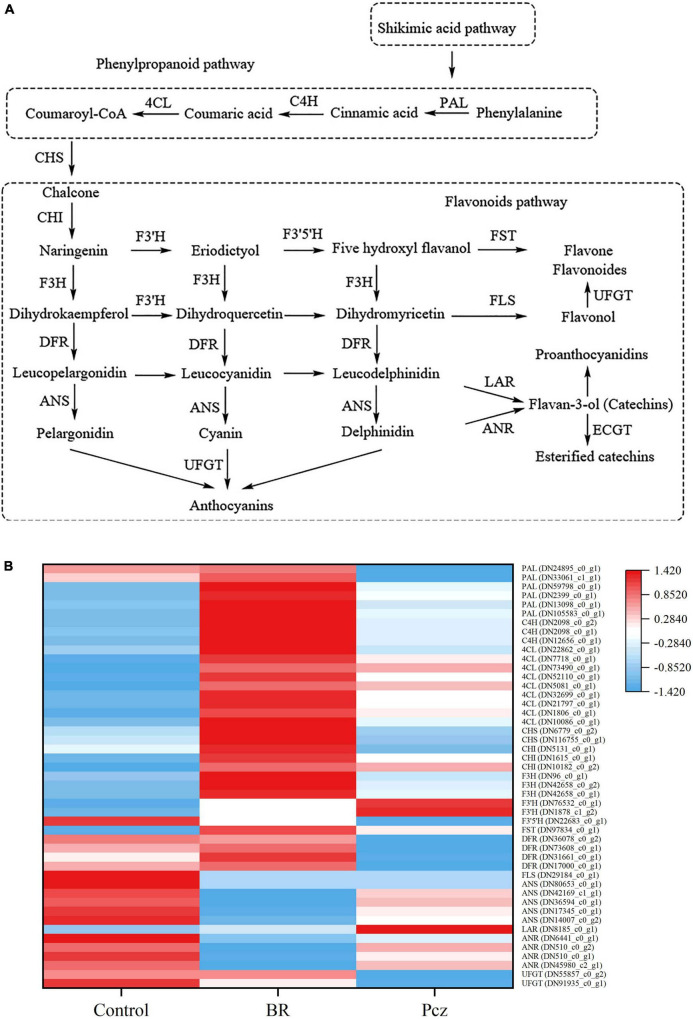
Analysis of differentially expressed levels related to flavonoid biosynthesis pathway in bulbil of *P. ternata* under BR and Pcz treatments. **(A)**, the flavonoid biosynthetic pathway. **(B)**, the expression levels related to the flavonoid biosynthetic pathway. Blue indicates a lower expression level, whereas red indicates a higher expression level. All the data showed the average mean of three biological replicates.

Functional annotation results from six databases showed forty-seven unigenes associated with the flavonoid biosynthesis pathway (Figure 7). In the flavonoid biosynthesis pathway, we identified that the phenylalnine ammonialyase (PAL, six genes), cinnamate 4-hydroxylase (C4H, three genes), 4-coumaroyl-CoA ligase (4CL, nine genes), chalcone synthase (CHS, two genes), chalcone isomerase (CHI, three genes), flavanone 3′-hydroxylase (F3H, three genes), flavonoid 3′-hydroxylase (F3′H, two genes), flavanol 4′-sulfotransferase (FST, one gene), and leucoanthocyanidin reductase (LAR, one gene) were higher expressed in BR-treated group. Application of BR improved the expression of flavonoid 3′, 5′-hydroxylase (F3′5′H, one gene), flavanol synthase (FLS, one gene), anthocyanidin synthase (ANS, five genes), and anthocyanidin reductase (ANR, four genes) genes. A total of nineteen unigenes encoding enzymes, including CHS (two genes), F3′5′H (one gene), dihydroflavonol 4-reductase (DFR, four genes), FLS (one gene), ANS (five genes), ANR (four genes), and UDP-glucose flavonoid 3-O-glucosyltransferase (UFGT, two genes), were lower expressed in Pcz treatment. Meanwhile, application of Pcz improved the expression of C4H (three genes), 4CL (nine genes), F3H (three genes), F3′H (two genes), FST (one gene), and LAR (one gene).

### Differentially expressed genes involved in the ascorbic acid biosynthesis

**FIGURE 8:**
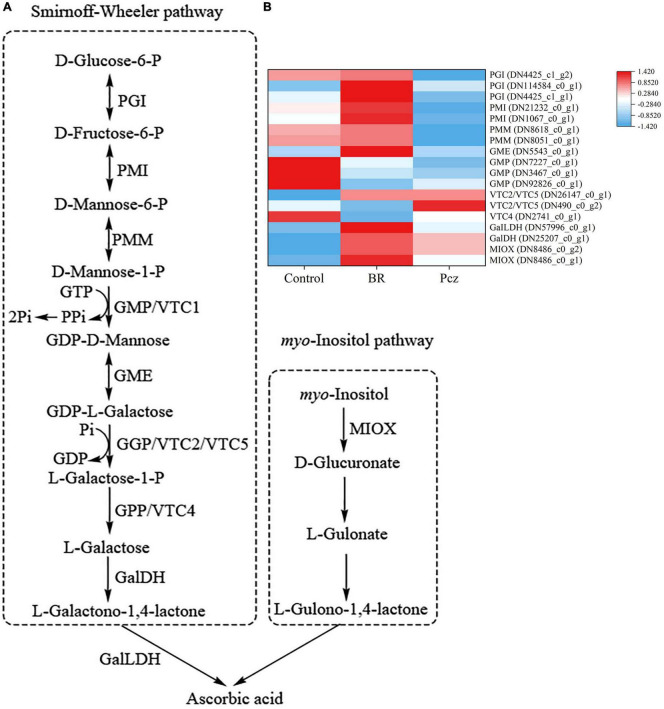
Analysis of differentially expressed levels related to the ascorbic acid pathway in bulbil of *P. ternata* under BR and Pcz treatments. **(A)**, the ascorbic acid biosynthetic pathway. **(B)**, the expression levels of genes related to the ascorbic acid biosynthetic pathway. Blue indicates a lower expression level, whereas red indicates a higher expression level. All the data showed the average mean of three biological replicates.

A total of seventeen unigenes encoding enzymes were assigned to the ASA biosynthesis based on the six databases (Figure 8). Among these genes, BR treatment had higher expression levels of phosphoglucose isomerase (PGI, three genes), phosphomannose isomerase (PMI, two genes), phosphomannomutase (PMM, two genes), GDP-d-mannose 3′, 5′-epimerase (GME, one gene), L-L-galactono-1,4-lactone dehydrogenase (GalLDH, one gene), l-galactose dehydrogenase (GalDH, one gene), and myo-inositol oxygenase (MIOX, two genes) and lower expression levels of GDP-d-mannose pyrophosphorylase (GMP, three genes) and l-galactose-1-P phosphatase (VTC4, one gene). Application of Pcz improved the expression of PMI (two genes), PMM (two genes), GMP (three genes), and VTC4 (one gene). Meanwhile, the expression of GDP-L-galactose phosphorylase (VTC2/VTC5, two genes), GalLDH (one gene), GalDH (one gene), and MIOX (two gene) were improved by applying Pcz.

### Differentially expressed genes involved in the glutathione biosynthesis

**FIGURE 9:**
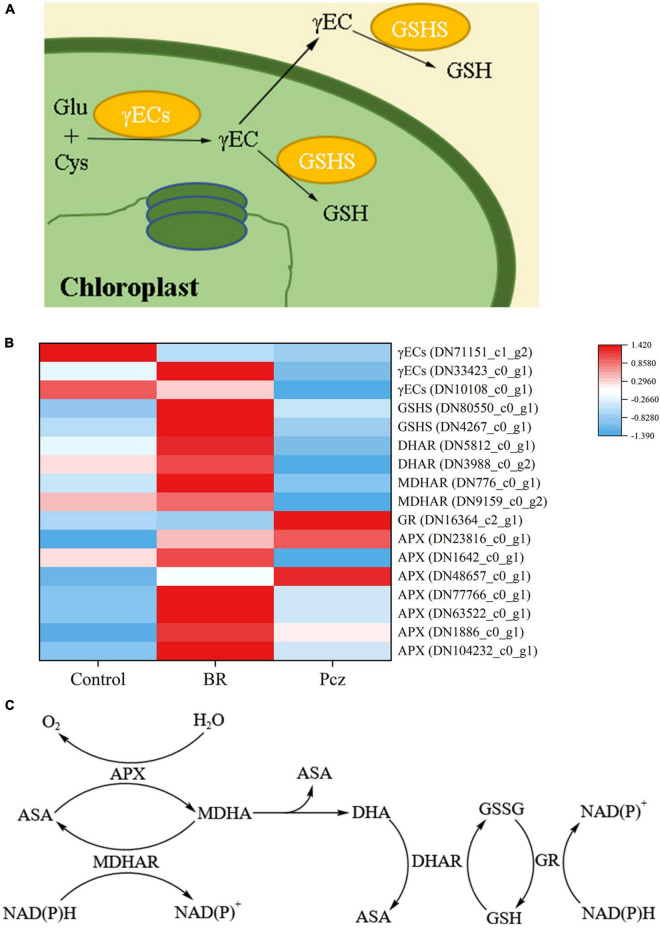
Analysis of differentially expressed levels related to glutathione and Ascorbate–Glutathione pathway in bulbil of *P. ternata* under BR and Pcz treatments. **(A)**, the glutathione biosynthetic pathway. **(B)**, the expression levels of genes related to the glutathione and Ascorbate-Glutathione pathway. **(C)**, the Ascorbate–Glutathione pathway. Blue indicates a lower expression level, whereas red indicates a higher expression level. All the data showed the average mean of three biological replicates.

As shown in Figure 9, the expression of GSH synthetase (GSHS, 2 genes), DHAR (2 genes), MDHAR (2 genes), and APX (7 genes) was enhanced by applying BR. However, the expression level of GR (1 gene) was downregulated by applying BR. Pcz treatment had lower expression levels of γ-glutamylcyteine synthetase (γECs, 3 genes), DHAR (2 genes), and MDHAR (2 genes) and had higher expression levels of GR (1 gene).

### qRT–PCR validation of the sequencing data

The expression of twenty-seven unigenes was verified by qRT**–**PCR. The expression trends of GalDH, PMI, F3’H, GSHS, AsA**–**GSH cycle, CHS, F3’5’H, UFGT, FST, CHI, F3H, 4CL, and C4L unigenes were highly consistent with the transcriptome data, and GaILDH, PMM, γECs, GMP, GME, VTC4, VTC2/VTC5, PGI, DFR, FLS, ANS, LAR, ANR, and PAL unigenes were compatible partially (Supplementary Figure 1). The R^2^ (COD) and Pearson’s correlation coefficient between the relative expression levels of RNA-Seq and qRT**–**PCR were 0.9529 and 0.9766, respectively (Supplementary Figure 2).

## Discussions

In the past few decades, with the development of transgenic technology, some advances have been made in improving plant resistance to abiotic stress by developing plants with high-antioxidant capacity. Overexpression of genes encoding substances related to the antioxidant system enhances the abiotic stress tolerance and antioxidant capacity of plants (Hasanuzzaman et al., 2020). In addition, many candidate genes associated with antioxidant capacity have been identified. It was believed that BR induced antioxidant defense systems in plants and activated the expression of numerous genes associated with plant responses to environmental stress (Gill et al., 2017). Exogenous application of BR is an effective alternative to genetic engineering in improving plant antioxidant capacity.

Flavonoids are important non-enzymatic antioxidant substances which contributes to the defense against abiotic stresses (Hichri et al., 2011). Both the hydroxyl groups present in the flavonoid structure and structural modifications, such as glycosylation, prenylation, and methylation, could be beneficial for the scavenging of ROS in plants (Ghassemi-Golezani et al., 2020). Flavonoid biosynthesis pathways have been well described in the last decades and have identified many key genes involved in the environmental stress responses. The possible biosynthesis pathway of flavonoid in bulbil of *P. ternata* was presented in Figure 7A (Wang et al., 2012). We investigated the response of the flavonoid biosynthesis pathway to applying BR under non-stress conditions. The PAL is a key enzyme for biosynthesis in plants, catalyzing the conversion of phenylalanine to trans-cinnamic acid. Therefore, it is very important for plant growth and tolerance to abiotic stress. Earlier research revealed that the expression level of PAL gene was in line with the changes in flavonoid content (Zhang et al., 2019). We found similar results with BR treatment increasing the total flavonoid content, and the expression levels of two PAL genes (DN24895_c0_g1 and DN33061_c1_g1) were increased by applying BR but were decreased by applying Pcz. The first step of the flavonoid biosynthesis pathway is the production of chalcone catalyzed by CHS from coumaroyl-CoA. Li et al. (1993) reported that inactivation of CHS genes reduced flavonoid accumulation in *Arabidopsis*. Overexpression of CHS genes enhanced the flavonoid content in *Silybum marianum* (Rahnama et al., 2013). We identified that two CHS genes (DN6779_c0_g2 and DN106755_c0_g1) were up-regulated in BR treatment but were down-regulated in Pcz treatment compared with the control. In addition, the expression of one CHI gene (DN5131_c0_g1) was enhanced by applying BR but was reduced by applying Pcz. Overexpression of the CHI gene caused an increased flavonols content in tomatoes (Muir et al., 2001). Ma et al. (2014) reported that CHS and CHI had critical roles in protecting wheat from drought stress. This result indicated that applying BR increased the flavonoid content by enhancing the expression levels of CHS and CHI genes. Thus, BR improved *P. ternata* tolerance to abiotic stress by increasing the flavonoid content. DFR catalyzes the conversion of dihydrokaempferol to leucopelargonidin and is a key enzyme in anthocyanins biosynthesis (Zhang et al., 2019). In the present study, the expression levels of three DFR genes (DN73608_c0_g1, DN31661_c0_g1, and DN17000_c0_g1) were improved by applying BR but were decreased by applying Pcz. Anthocyanin biosynthesis in bayberry was regulated by substrate competition between FLS and DFR (Shi et al., 2018), suggesting that BR treatment increased the anthocyanins content by enhancing the DFR genes expression and decreasing the FLS genes (DN29184_c0_g1) expression. UFGT contributes to the chemical diversity of flavonol and catalyzes the conversion of pelargonidin to anthocyanins (Lai et al., 2015). In the present study, Pcz treatment decreased the expression level of one UFGT gene (DN55857_c0_g1), which was not affected by applying BR. This result suggested that BR has an essential role in UFGT gene expression, and inhibition of endogenous BR synthesis decreased UFGT gene expression.

Ascorbic acid, as a low-molecular-weight non-enzymatic antioxidant, is able to directly scavenge ROS in plants (Kim et al., 2017). Previous studies showed that the accumulation of ASA improved abiotic stress tolerance in strawberries (Galli et al., 2018) and potatoes (Upadhyaya et al., 2009). There are two ways to enhance the accumulation of ASA, including the ASA biosynthesis pathway and recycling pathways (Wang et al., 2013). In the ASA biosynthesis, the main pathway of Smirnoff-wheeler and alternative pathway of myo-inositol were shown in Figure 8A (Ortiz-Espín et al., 2017; Ivanov Kavkova et al., 2018). We identified two PGI genes (DN4425_c0_g2 and DN4425_c0_g1), two PMI genes (DN21232_c0_g1 and DN1067_c0_g1), and two PMM genes (DN8618_c0_g2 and DN8051_c0_g1) were higher expressed in BR treatment but were lower expressed in Pcz treatment. Moreover, the expression of the GME gene (DN5543_c0_g1) was increased by applying BR, which was not affected by applying Pcz. Our results suggested that applying BR improved the ASA content by increasing the expression levels of PGI, PMI, PMM, and GME genes. Similar results were also found that the PMI gene expression was positively correlated with ASA content, and silencing of the PMI gene decreased the ASA content by 50% in *Arabidopsis* (Maruta et al., 2008). Qian et al. (2007) reported that overexpression of PMM genes increased the ASA levels by 20–50% in *Nicotiana benthamiana* and *Arabidopsis*. The PMM genes were associated with the increased ASA content in tobacco, but may not be a critical factor in ASA biosynthesis (Badejo et al., 2009). Silencing of the GME gene using RNAi reduced ASA content in tomatoes (Gilbert et al., 2009). In the present study, ASA content was increased by applying Pcz, which was not affected by applying BR. A research conducted by Dowdle et al. (2007) showed that exogenous ASA suppressed the expression level of the VTC2 gene in *Arabidopsis*. In this study, the expression level of VTC2/VTC5 (DN490_c0_g2) was reduced by applying BR but was enhanced by applying Pcz, indicating that exogenous application of BR reduced the expression of the VTC2 gene and thus inhibited the ASA biosynthesis.

As shown in Figure 9A, GSH biosynthesis involves two enzymatic steps (Hasanuzzaman et al., 2019). The expression of one γECs (DN33423_c0_g1) and two GSHS (DN80550_c0_g1 and DN4267_c0_g1) genes were enhanced by applying BR but were reduced by applying Pcz (except for DN80550_c0_g1), indicating that applying Pcz decreased the GSH biosynthesis by decreasing the expression levels of γECs and GSHS genes. It has been reported that overexpression of γECs and GSHS increased the GSH content in plants, and the effect of the γECs gene on GSH content was greater than that of the GSHS gene (Noctor et al., 2012). However, our result showed that BR and Pcz treatments significantly decreased the GSH content. This was probably because the BR application reduced the GR activity and enhanced the DHAR activity (Figures 2D, 9B). Thus, BR treatment decreased GSH content in the ASA–GSH cycle. The ASA–GSH cycle is a vital process for the scavenging of ROS, which is mainly detoxification of H_2_O_2_ in plant cells, involving MDHAR, DHAR, APX, and GR enzymes (Hasanuzzaman et al., 2019). The expression of six APX (DN23816_c0_g1, DN1642_c0_g1, DN77766_c0_g1, DN63522_c0_g1, DN1886_c0_g1, and DN104232_c0_g1) genes were increased by BR and Pcz treatment, which were associated with the increased APX activity. A similar study reported that 24-epibrassinolide treatment significantly increased the APX activity and enhanced the expression level of the APX gene in *Cucumis sativus* (Ahammed et al., 2017; Guedes et al., 2021). The GR activity and expression level (DN16364_c2_g1) were reduced by applying BR but were enhanced by applying Pcz. A research conducted by Gill et al. (2017) showed that the application of BR (0.1 mg l^–1^) had no significant effects on GR activity in the roots of barley. This was probably because the exogenous application of BR increased GSH biosynthesis and decreased GSH content in the ASA–GSH cycle. The expression of two MADAR (DN776_c0_g1 and DN9159_c0_g2) and two DHAR (DN5812_c0_g1 and DN3988_c0_g2) genes was enhanced by applying BR but was reduced by applying Pcz. These results suggested that applying BR was beneficial to ASA recycling and GSH oxidation in the ASA–GSH pathway. Superoxide dismutase and POD are essential components of the enzymatic antioxidant system. In this paper, there was no significant effect of applying BR and Pcz on the SOD activity. Similar results were also found in the study of Kaya et al. (2020b). Previous studies showed that the application of BR (0.1 mg l^–1^) raised the POD activity in the roots of barley (Gill et al., 2017). In the present study, the POD activity was increased by applying BR but was decreased by applying Pcz.

## Conclusion

Exogenous application of BR improved antioxidant capacity in bulbil of *P. ternata* under non-stress conditions. The antioxidant capacity was also reflected in the increased DPPH radical scavenging activity and reduced MDA content. We observed that the BR treatment upregulated the expression levels of enzymes in the flavonoid, ASA, and GSH biosynthesis pathways to increase their content. In addition, the ASA–GSH cycle and antioxidant enzyme activity were also promoted by applying BR. These results were in line with the previous hypothesis that applying BR increases the antioxidant capacity by enhancing the enzymatic and non-enzymatic defense systems under non-stress conditions. Finally, our results have provided molecular evidence about the role of BR in transcriptional regulation of antioxidant-related genes in P. ternata under non-stressful condition.

## Data availability statement

The original contributions presented in the study are publicly available. This data can be found here: https://www.ncbi.nlm.nih.gov/, PRJNA719943.

## Author contributions

CG, JC, and XY designed the experiments and contributed to writing and revising the manuscript. CG, YC, MW, YD, and DW performed the experiments. CG analyzed the data. JC and XY supervised the study. All the authors contributed to the article and approved the submitted version.
