# Supplementary material for: Exogenous brassinolide improves the antioxidant capacity of Pinellia ternata by enhancing the enzymatic and nonenzymatic defense systems under non-stress conditions
Source: Front Plant Sci. 2022 Jul 25;13:917301. doi: 10.3389/fpls.2022.917301 (PMC9358693; doi:10.3389/fpls.2022.917301)
Supplement: Supplementary file 1 [file Data_Sheet_1.DOCX]

Supplementary Table 1 Primers used for qRT-PCR

| Gene | Forward (5'-3') | Rearward (5'-3') |
| --- | --- | --- |
| C4L | GTAGTCCTTGAACAGCTGGAG | CAGAGCTTCGACTACAACTACG |
| CHS | AGACAAACACCAGACACTCG | CGTCGCCTTCCATAGCTG |
| CHI | TCTCTTTCCTTGTTCCCTGC | CGCCTACCACTCCAACAC |
| F3H | TGTTCTTGGTCTAGGTTGCG | GGTGGCATCGTTCCTCTG |
| F3'H | CCTACTTGGGCCGTTTATCTAG | ATGAGGTTGAGATTGCCGAC |
| F3'5'H | TCTTGCTCAATATCCTGTCGC | GGTTCGATCTGCAAGGAGTC |
| FST | GACTCTTCAGATATCCCGTGG | CGAGTCTGGACAAGGTCATC |
| DFR | AGCTAGCAGGTATGTGGTTG | TCTCTAATTACAAGCTCGCCG |
| FLS | AAGTTTGAAGGATACTCTGGCC | GTGGGACCTGGGATTTATGG |
| ANS | TCCTTCACATCACCCAAACAC | GTTCTGGGTTGGGAGATACTTG |
| LAR | CCGACGGCAAAAGGAATATTG | GTTGAACCCAGAATCTTGCAG |
| ANR | GTGGTTCTGGACTGTGGAATAG | ATGAGGTTGGCGAGACATG |
| UFGT | TCCAGCTCGTAGAATGTGTTC | CGCCGGAATTTTGAACAGAC |
| PGI | CATTCTACCAGCTCATCCACC | ATGCAGACCTTTCCGTATGG |
| PMI | CTCGTCCTCTTGCTCTTCC | CCCCAGTCGTAAGTTTGCAC |
| PMM | TTCACCCAGCAAAGTCAGAG | CATTTTGTCCGACCCAAACAC |
| GMP | GCTCTTCCTGTCCTCTTTCG | CTGTCCATGTCCGTCTTCAG |
| GME | CCTCAGACCATCCTTCAGTTTC | AGTTTTGAGGACAGGAAGCTC |
| VTC2/5 | CCCTCTTAATGGTCAGCATCC | GGCACTTTTGTTTCTCGCAG |
| VTC4 | GCCGCTACTTTCCTAGATGTG | TCCGTAAGTATTTCTCAAACCCTG |
| GalDH | GGATATTCCCCTACGTGCTC | TGTCGTTGATGCTGTAGTGG |
| GaILDH | TGAGATGCTTTACCCGTACTTC | ACAGTTGTGGTTGTACGGTG |
| γECs | TGTGAGAGTAACGAAGGTGATG | CCAGTCACAGGAACCTATTGAG |
| GSHS | GTCCAAAACTGCGAATCCTG | CGGAACTGGGTGTATATGGTG |
| AsA-GSH | GTGAATAGGATGTAGGGTGCTG | AGGAATGGAGCAACTGGAAG |





Supplementary Figure 1 Comparison of the expression patterns of genes involved in the flavonoids, ascorbic acid, glutathione, and ASA-GAH pathway in bulbil of *P. ternata* under control, BR, and Pcz treatments by qRT-PCR and RNA-seq. values are means of three replicates ± SE.





Supplementary Figure 2 correlation plot between RNA-seq and qRT-PCR for the relative expression of unigenes.
